# Particle-antiparticle duality and fractionalization of topological chiral solitons

**DOI:** 10.1038/s41598-020-80085-8

**Published:** 2021-01-13

**Authors:** Chang-geun Oh, Sang-Hoon Han, Seung-Gyo Jeong, Tae-Hwan Kim, Sangmo Cheon

**Affiliations:** 1 Research Institute for Natural Sciences, Hanyang University, Seoul, 04763 Korea; 2 Department of Physics, Hanyang University, Seoul, 04763 Korea; 3 Department of Physics, Pohang University of Science and Technology (POSTECH), Pohang, 37673 Korea

**Keywords:** Electronic properties and materials, Topological matter, Topological defects

## Abstract

Although a prototypical Su–Schrieffer–Heeger (SSH) soliton exhibits various important topological concepts including particle-antiparticle (PA) symmetry and fractional fermion charges, there have been only few advances in exploring such properties of topological solitons beyond the SSH model. Here, by considering a chirally extended double-Peierls-chain model, we demonstrate novel PA duality and fractional charge *e*/2 of topological chiral solitons even under the chiral symmetry breaking. This provides a counterexample to the belief that chiral symmetry is necessary for such PA relation and fractionalization of topological solitons in a time-reversal invariant topological system. Furthermore, we discover that topological chiral solitons are re-fractionalized into two subsolitons which also satisfy the PA duality. As a result, such dualities and fractionalizations support the topological $\mathbb {Z}_4$ algebraic structures. Our findings will inspire researches seeking feasible and promising topological systems, which may lead to new practical applications such as solitronics.

## Introduction

Topological solitons are ubiquitous in nature and have been widely investigated as exotic extended objects in the various systems^1^. In quantum topological physics, the famous Su-Schrieffer-Heeger (SSH) soliton manifests a variety of important concepts including Thouless topological charge pumping, fractional charge, particle-antiparticle (PA) symmetry and spin-charge separation^2–7^. Such exotic properties and potential applications have stimulated many studies: conducting polymers^7^, diatomic chain model^8^, fractionalized domain wall excitations in 1D chain and wire^9,10^, acoustic experimental system^11^, realization of Zak phase and topological charge pumping in the cold atom system^12–14^, topological photonic crystal nanocavity lasers using SSH edge mode^15–17^, artificially engineered SSH lattices^18,19^, and topological quaternary operation using chiral soliton^20,21^. Among such exotic properties, PA symmetry and fractionalization are essential to understand not only the pair creation but also topological properties of topological solitons. Due to its PA symmetry, an SSH soliton is its own antisoliton (antiparticle) having a zero energy state with a fractional fermion number $\pm \frac{1}{2}$ in contrast to conventional fermion excitations in solids (electrons or holes)^2,4^. Though there were several attempts to extend SSH model (for example, considering more atoms in a unit cell, interactions beyond nearest neighbor, spin-orbit coupling, or nonsymmorphic symmetries)^8,9,22–28^, there have been only few substantial studies seeking PA symmetry and fractionalization beyond the SSH model.

Here we consider a chirally extended SSH model, double-Peierls-chain model, realized in indium atomic chains on a silicon substrate^29,30^. The double-chain model possesses not only $\mathbb {Z}_4$ symmetry and topological chiral solitons having new chiral degree of freedom^20^ but also chiral switching between such solitons^21^. In this model, we find novel particle-antiparticle duality and the fractional charge *e*/2 of topological chiral solitons even in the presence of chiral symmetry breaking. We find the possible PA dualities and symmetries among topological chiral solitons using two classes of fundamental charge-conjugation operators combined with nonsymmorphic operators. The first class gives the distinguishable PA duality between right-chiral (RC-) and left-chiral (LC-) solitons, which allows that RC- and LC-solitons with the opposite quantum numbers can be pair created and pair annihilated; the second one provides the self PA duality to an achiral (AC-) soliton being its own antiparticle like Majorana fermions^31^. Furthermore, we demonstrate that each chiral soliton is re-fractionalized into two subsolitons, which we will refer to as “2nd fractionalization.” Through the 2nd fractionalization, RC- and LC-solitons are divided into primary and induced subsoliton states while AC-solitons are split into bonding and antibonding subsoliton states. Thus, when a pair of solitons is created, a subsoliton quartet emerges from a groundstate for all types of chiral solitons, which has not been observed or even proposed in any physical systems.

## Results and discussion

**Figure 1:**
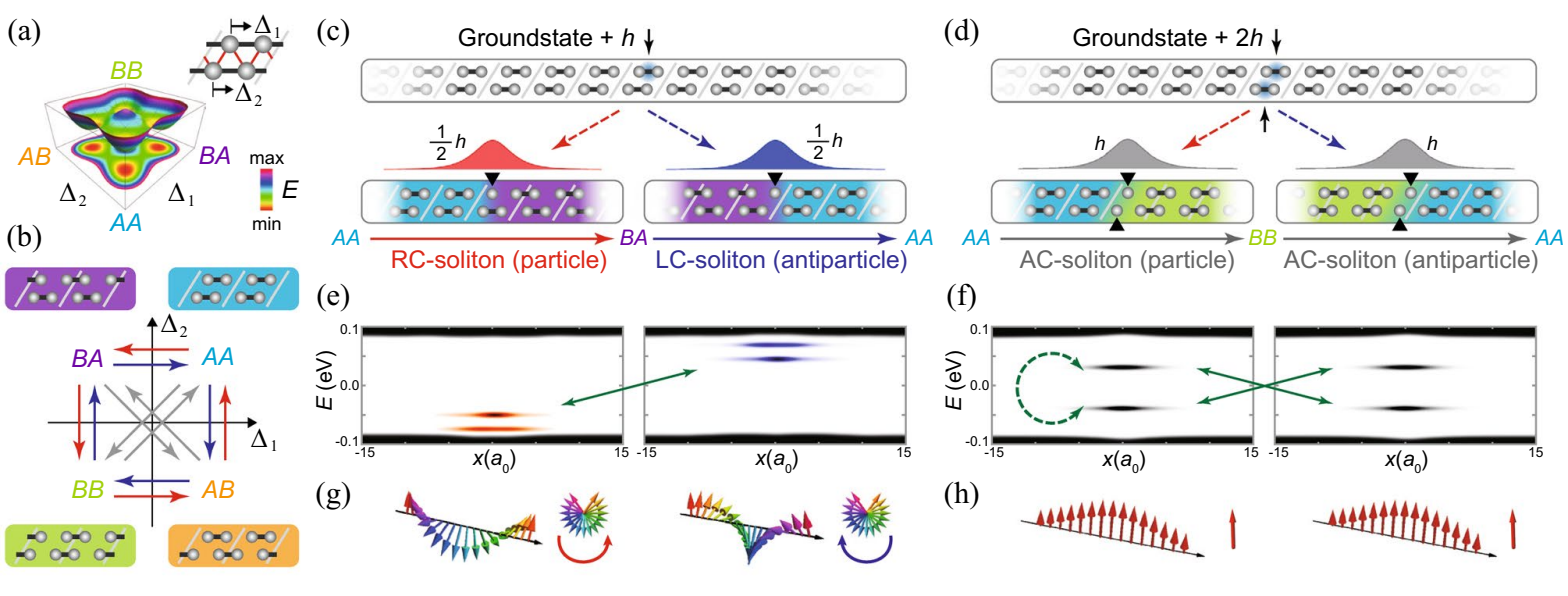
Particle-antiparticle (PA) duality of topological solitons in the double-Peierls-chain model. **(a)** Total energy (left diagram) of double-Peierls-chain model (right diagram) as a function of order parameters $\Delta _i$ ($i=1,2$), where $\Delta _i$ is the atomic dimerization displacement of the *i*-th chain. The model is composed of four atoms in a unit cell with interchain coupling (red lines). (**b**) Atomic configurations of four groundstates and 12 solitons (interpolating two of groundstates) in the order-parameter space. Right-chiral (RC), left-chiral (LC), and achiral (AC) solitons are represented by red, blue, and gray arrows, respectively. (**c**, **d**) Pair-creation of (**c**) chiral and (**d**) achiral solitons by adding a hole *h* or two holes 2*h* to a groundstate. Below each soliton profile, the corresponding atomic configuration is demonstrated and black triangles show soliton centers (unpaired atoms or kink positions). (**e**, **f**) Spatially resolved local densities of states (LDOS’s) of (**e**) RC-, LC-, and (**f**) AC-solitons. In each energy gap, RC-, LC-, and AC-subsoliton states are represented by red, blue, and gray colors. Solid green arrows indicate the PA partners (**e**) between RC- and LC-subsoliton states and (**f**) between AC-subsoliton states. Dashed green arrow represent that each AC-subsoliton is its own PA partner. (**g**, **h**) Spatial distribution of pseudospin vectors of (**g**) RC-, LC-, and (**h**) AC-solitons along the chain. Pseudospin vectors of RC- and LC-subsolitons rotate oppositely along the chain while those of AC-subsolitons do not rotate.

We investigate the PA duality and fractionalization of topological solitons in the double-Peierls-chain model^20^ which has AI symmetry^32^ (preserved time-reversal symmetry and broken chiral symmetry) and four degenerate groundstates in the order-parameter spaces (Fig. 1a). There are twelve topological chiral solitons that connect two of four groundstates and the interchain coupling induces dynamical chiral symmetry breaking leading to three types of topological chiral solitons: RC-, LC-, and AC-solitons (Fig. 1b).

RC- and LC-solitons are pair-created from a groundstate by adding a hole *h* while equally dividing that hole (Fig. 1c). However, the broken charge-conjugation symmetry makes RC- and LC-solitons distinguishable in various ways while the soliton and antisoliton in the SSH model are indistinguishable. The calculated spatially-resolved local densities of states (LDOS’s) of RC- and LC-solitons are located oppositely with respect to $E=0$ and their pseudospin vectors rotate oppositely to each other as shown in Fig. 1e,g. These findings strongly imply the PA duality between RC- and LC-solitons. By the way, two subsoliton states appear in both RC- and LC-soliton spectra. This is the result of “2nd fractionalization,” which will be proved later.

On the other hand, two AC-solitons (an AC-soliton and an anti-AC-soliton) having a hole per soliton are generated through a pair production from a groundstate when two bonding electrons are removed (Fig. 1d). As shown in Fig. 1f, the calculated LDOS’s of an AC-soliton and an anti-AC-soliton are both identical and symmetric each other (solid arrows) implying PA duality. Similar to the “2nd fractionalization” of RC- and LC-solitons, two AC-subsoliton states—bonding and antibonding states—appear in the gap of both an AC-soliton and an anti-AC-soliton (Fig. 1f). The calculated LDOS’s of the bonding and antibonding states are located oppositely with respect to the $E=0$ (dashed arrow) implying self PA duality. Moreover, the pseudospins of AC-solitons do not rotate (Fig. 1h) because an AC soliton does not have chirality^20^. Hence, we cannot distinguish an AC-soliton from its anti-AC-soliton with the same charges, energy spectra, and pseudospin vectors (Fig. 1d,f,h) like a pair of SSH soliton and antisoliton. These observations strongly suggest that each AC-soliton should show PA dualities with itself as well as with its anti-AC-soliton while a pair of RC- and LC-solitons exhibits PA duality.

**Figure 2:**
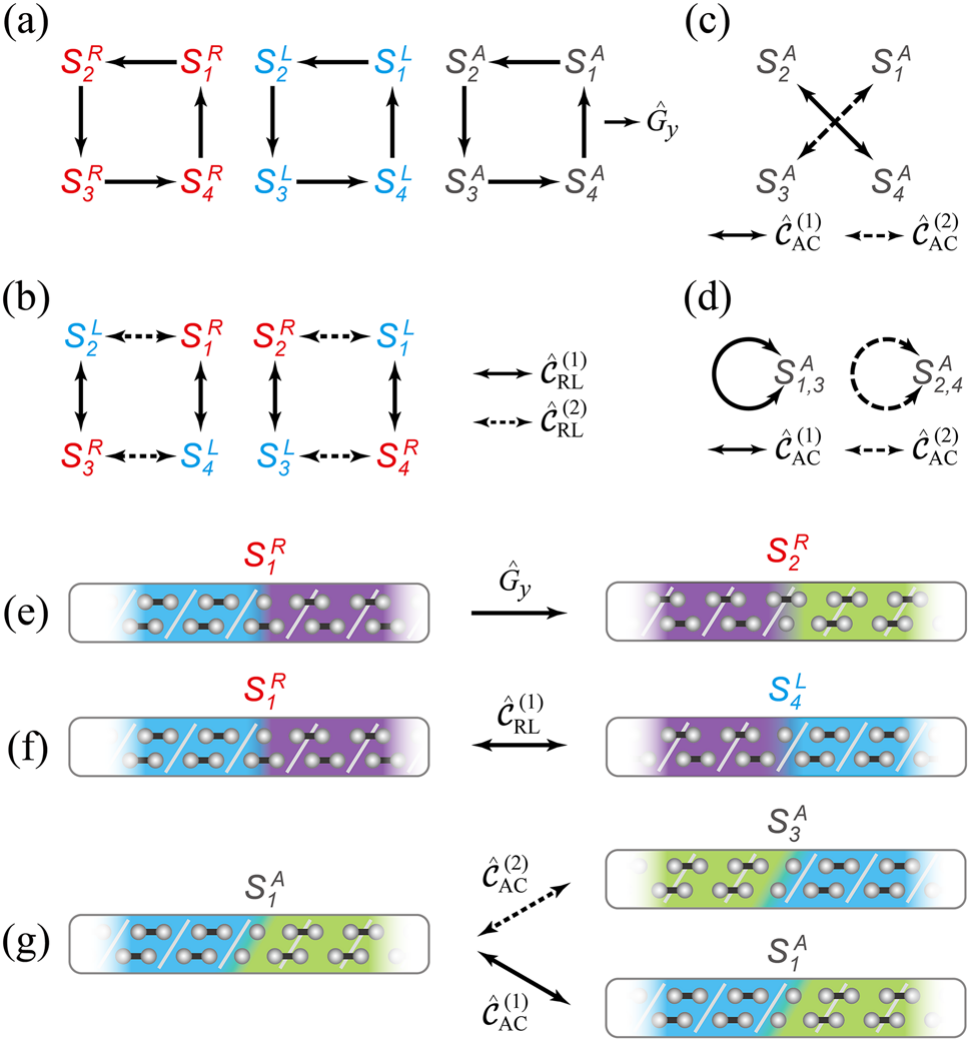
Symmetry transformations among topological solitons. **(a)** Equivalent transformations among the same types of solitons under the glide reflection $\hat{G}_y$. The cyclic group (or $\mathbb {Z}_4$ symmetry group) among solitons are formed for (red) RC-, (blue) LC-, and (black) AC-solitons. **(b)** PA duality transformations between RC- and LC-solitons under the nonsymmorphic charge-conjugation operation $\hat{\mathcal {C} }_{\text {RL}}^{(i)}$ ($i = 1, 2$). (**c**) PA duality transformations among AC-solitons. **(d)** Self PA duality transformations of AC-solitons. In (**c**) and (**d**), the same nonsymmorphic charge-conjugation operator $\hat{\mathcal {C} }_{\text {AC}}^{(i)}$ ($i = 1, 2$) gives different transformations: in (**c**), two AC-solitons transform to each other while each AC-soliton transforms into itself in (**d**). Here, $S^R_i$, $S^L_i$, and $S^A_i$ denote specific RC-, LC-, and AC-solitons (see Table S1 in Supplementary information). **(e)**–**(g)** Real-space illustrations of the representative transformation among solitons: (**e**) equivalent transformation between two RC-solitons; (**f**) PA duality transformation between RC- and LC-solitons; (**g**) (upper) PA duality transformation between AC-solitons and (lower) self PA duality transformation in an AC-soliton.

To prove the various PA dualities among RC-, LC-, and AC-solitons, we perform a symmetry analysis in the framework of the low energy effective theory using nonsymmorphic operators. Since there is no chiral symmetry and the conventional charge-conjugation operator does not give any PA relation in this model, we find three classes of nonsymmorphic operators that give significant relations among solitons: a glide reflection operator ($\hat{G}_y$) and two nonsymmorphic charge-conjugation operators ($\hat{\mathcal {C}}_{\text {RL}}^{(i)}$ and $\hat{\mathcal {C}}_{\text {AC}}^{(i)}$). See Section S2 for details in Supplementary information.

**Figure 3:**
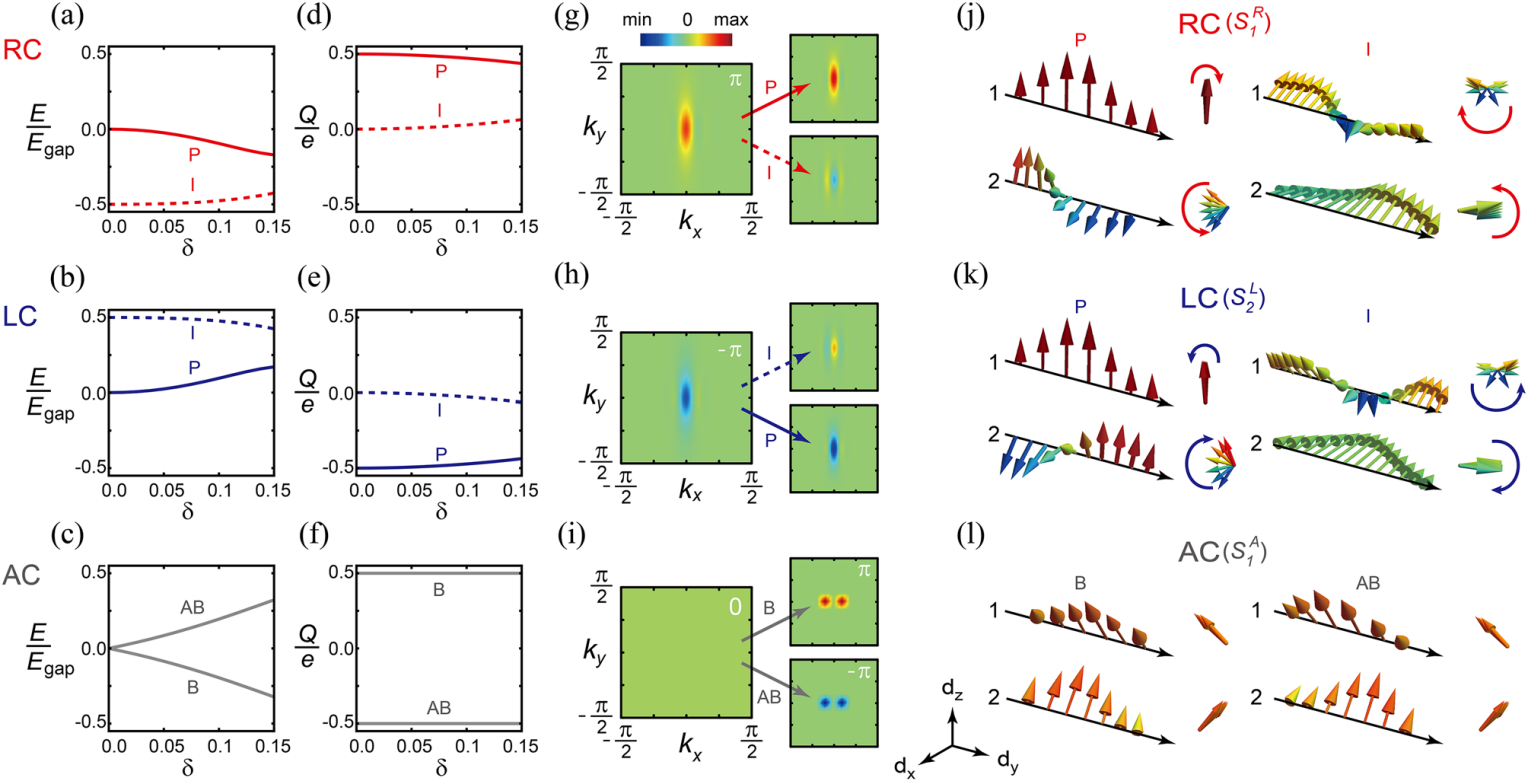
Fractionalized topological solitons and their PA dualities. (**a**–**c**) Spectra of fractionalized solitons with respect to the interchain coupling strength $\delta$ for RC-, LC-, and AC-solitons. In (**a**) and (**b**), the primary (P) and induced (I) subsoliton states are represented by solid and dashed lines, respectively. In (**c**), bonding (B) and antibonding (AB) subsoliton states are indicated by gray lines. The soliton spectra are normalized by the energy gap $E_{\text {gap}}$ for each $\delta$. (**d**–**f**) Topological charges (or equivalently electron numbers) of subsolitons with respect to $\delta$. Topological charges are normalized by the electron charge *e*. In each plot, the sum of topological charges of two subsolitons are the quantized: (**d**) *e*/2, (**e**) $-e/2$, and (**f**) 0. (**g**–**i**) Fractionalized Berry curvatures for (**g**) RC-, (**h**) LC-, and (**i**) AC-solitons ($\delta =0.1$). The total Berry curvature (left) is divided into two fractionalized Berry curvatures (right). The integrated value is indicated at the upper right corner in each plot. (**j**–**l**) Spatially distributed pseudospin vectors for (**j**) RC-, (**k**) LC-, and (**l**) AC-subsolitons along each chain. Pseudospin vectors of primary (left diagram) and induced (right diagram) subsoliton states are plotted for (**j**) RC- and (**k**) LC-solitons. Compared with the RC-soliton, each pseudospin vector for the LC-soliton rotates oppositely due to PA duality. In (**l**), the pseudospin vectors of bonding states in one chain (left diagram) are related to those of antibonding states in the other chain (right diagram) by reversing *x*-component due to self PA duality (see Section S5 in Supplementary information).

We now discuss the main results of symmetry analysis. First, $\hat{G}_y$ establishes equivalent relations among solitons having the same chirality (Fig. 2a), which guarantees the same physical properties (energy spectra, soliton lengths, and topological charges) and $\mathbb {Z}_4$ cyclic properties among solitons. Second, $\hat{\mathcal {C}}_{\text {RL}}^{(i)}$ transforms RC-solitons to LC-solitons and vice versa (Fig. 2b), indicating that they are in PA duality. This operator assures that the quantum wavefunctions of RC- and LC-solitons are explicitly paired; $\Psi _{\text {RC}} = \hat{\mathcal {C}}_{\text {RL}}^{(i)} \Psi _{\text {LC}}$. Like the conventional charge-conjugation operator $\hat{\mathcal {C}}$, such PA duality supports that RC- and LC-solitons have opposite energy spectra and pseudospin vectors (Fig. 1e,g) as well as opposite topological charges (Fig. 3d,e). In this sense, two successive operations of $\hat{\mathcal {C}}_{\text {RL}}^{(i)}$ bring a soliton to its original state due to $(\hat{\mathcal {C}}_{\text {RL}}^{(i)})^2=1$ implying $\mathbb {Z}_2$ duality. It is noteworthy that this PA duality confirms the experimentally measured RC- and LC-soliton spectra^20,21^. Third, we find that an AC-soliton and its anti-AC-soliton are transformed to each other under $\hat{\mathcal {C}}_{\text {AC}}^{(i)}$ indicating PA duality (Fig. 2c). Together with the equivalent relation between AC-solitons (Fig. 2a), this PA duality of AC-solitons naturally leads to self PA duality (Fig. 2d), indicating that each AC-soliton becomes its own antisoliton similar to a Majorana fermion. Hence, within an AC-soliton, $\hat{\mathcal {C}}_{\text {AC}}^{(i)}$ exchanges the bonding and antibonding subsoliton states as shown in Fig. 1f. While the SSH soliton is a self-charge-conjugate state ($\Psi _{\text {S}} = \Psi _{\text {S}}^*$, where $\Psi _{\text {S}}$ is the SSH soliton wavefunction^4^), the AC-soliton is unexpectedly a pseudo-self-charge-conjugate state; $\Psi _{\text {AC}} = U \Psi _{\text {AC}}^*$, where *U* is an unitary operator and $\Psi _{\text {AC}}$ is an AC-soliton wavefunction including both bonding and antibonding subsoliton states. We also find that the geometrical operations of three types of nonsymmorphic operators in the real space consistently prove the relations among solitons (Fig. 2e–g). Based on this symmetry analysis, we further confirm that dualities are well matched with the chiral characters of chiral solitons; PA dual partners have opposite chirality while self PA duality indicates achiral.

Beyond the fractionalization of the SSH soliton, we find that RC- and LC-solitons are fractionalized once more. Both RC- and LC-solitons are split into primary and induced subsolitons through “2nd fractionalization” as shown in the spectra (Fig. 3a,b). The primary subsoliton resides mainly in a chain having a kink (black triangles in Fig. 1c) while the induced one does in the other chain (see Fig. S2 in Supplementary information). If a primary subsoliton in one chain is generated, then an effective mass inversion simultaneously takes place in the other chain leading to an induced subsoliton^20^. “2nd fractionalization” is the process of dividing the one-half fermion—split by “1st fractionalization” in the pair creation process (Fig. 1c)—into two fractional fermions possessed by two subsolitons (Fig. 3d,e). Naturally, the total fermion number of two subsolitons should be conserved with $\pm \frac{1}{2}$.

We calculate the topological charges of subsolitons with respect to the strength of the interchain coupling $\delta$ by using Goldstone-Wilczek formula and the low energy effective quantum field theory^33^. For an RC-soliton, the calculated fermion number for the primary and induced subsolitons are given by $N_{\text {P}}^{\text {RC}} = -\frac{1}{2} -x + n_{\text {occ}}$ and $N_{\text {I}}^{\text {RC}} = x$, respectively. For an LC-soliton, $N_{\text {P}}^{\text {LC}} = -\frac{1}{2} + x + n_{\text {occ}}$, $N_{\text {I}}^{\text {LC}} = - x$. $n_{\text {occ}}$ (0 or 1) is the occupation number of each primary subsoliton state. The opposite signs of *x* between RC- and LC solitons are the result of PA duality. Here, $x = \frac{\delta ^2 t_0^2}{\pi \Delta _0^2}$ is obtained up to leading order, where $t_0$ and $\Delta _0$ are the hopping integral and dimerization displacement (see Section S3 for details in Supplementary information). To see the PA duality more physically, we consider the Fermi-level ($E=0$) and a neutralized system, which implies that the primary subsoliton state of RC-soliton (LC-soliton) is filled (empty). Then, the electrical charges for the primary and induced subsolitons of an RC-soliton are given by $Q_{\text {P}}^{\text {RC}} = e \left( \frac{1}{2} -x \right)$, $Q_{\text {I}}^{\text {RC}} = e x$. For an LC-soliton, $Q_{\text {P}}^{\text {LC}} = - Q_{\text {P}}^{\text {RC}}$, $Q_{\text {I}}^{\text {LC}} = - Q_{\text {I}}^{\text {RC}}$, which explicitly shows the opposite electrical charges of a PA pair. Analytically, the charge conservation $Q_{\text {P}}^{\text {RC/LC}} + Q_{\text {I}}^{\text {RC/LC}} = \pm \frac{e}{2}$ is confirmed (Fig. 3d,e). Thus, the RC- and LC-solitons behave as a half electron and a half hole, respectively.

We confirm that the corresponding primary (induced) subsolitons for RC- and LC-solitons satisfy their own PA duality in their energy spectra. Figure 3a and b show the analytically calculated subsoliton spectra with respect to the interchain coupling strength $\delta$. For an RC-soliton, with increasing $\delta$, the energy state of the primary (induced) subsoliton decreases (increases) as shown in Fig. 3a. For an LC-soliton, the spectra of subsolitons show opposite behavior comparing with the RC-soliton (Fig. 3b) implying PA duality. Note that the level crossing between primary and induced subsolitons are observed for larger interchain coupling strength ($\delta \approx 0.2$). However, when the interchain coupling strength is large, the low-energy effective Hamiltonian description no longer works and hence nature of the primary and induced subsolitons cannot be described within the low-energy effective theory. Thus, the study about the effect of the strong interchain coupling on the topological solitons and their topological properties will be a future work.

To see the “2nd fractionalization” in the momentum space, we consider an adiabatic evolution from one to another groundstate, which corresponds to transporting a soliton very slowly. By taking the time-evolution as an extra momentum^34^, we calculate Berry curvatures of RC- and LC-solitons. See Section S4 in Supplementary information for details. The total Berry curvatures of RC- and LC-solitons are split into the fractionalized Berry curvatures of RC- and LC-subsolitons, respectively (Fig. 3g,h). Moreover, the fractionalized Berry curvatures of primary (induced) subsolitons for RC- and LC-solitons have opposite signs, which implies PA duality between subsolitons. Note that the fractionalized charges obtained by this fractionalized Berry curvature are consistent with those obtained by the Goldstone-Wilczek method. The quantum wavefunctions of each subsoliton state also manifest PA duality in real space as the pseudospin vectors of primary (induced) subsolitons for RC- and LC-solitons rotate oppositely (Fig. 3j,k).

For an AC-soliton, two identical SSH solitons, which are created by “1st fractionalization” having a half fermion in both chains, are fractionalized into the hybridized bonding and antibonding subsoliton states due to the interchain coupling (Fig. 1f). Hence, the fermion numbers of bonding and antibonding subsoliton states of an AC-soliton are divided as $N_{\text {B}} = -\frac{1}{2} + n_{\text {occ}}$ and $N_{\text {AB}} = -\frac{1}{2} + n_{\text {occ}}$, respectively. Likewise, due to the self PA duality, the electrical charges of such bonding and antibonding subsoliton states are opposite regardless of the interchain coupling when the system is neutralized; $Q_{\text {B}} = - Q_{\text {AB}} = e/2$ (Fig. 3f). This finding is consistent with the numerically obtained fractionalized Berry curvatures; the integrated values are $\pi$ and $-\pi$ for bonding and antibonding subsoliton states, respectively (Fig. 3i). The pseudospin vectors of the fractionalized bonding and antibonding AC-subsoliton states also respect to the self PA duality in the real space (Fig. 3l), so do the soliton wavefunctions (see Fig. S2c in Supplementary information).

**Figure 4:**
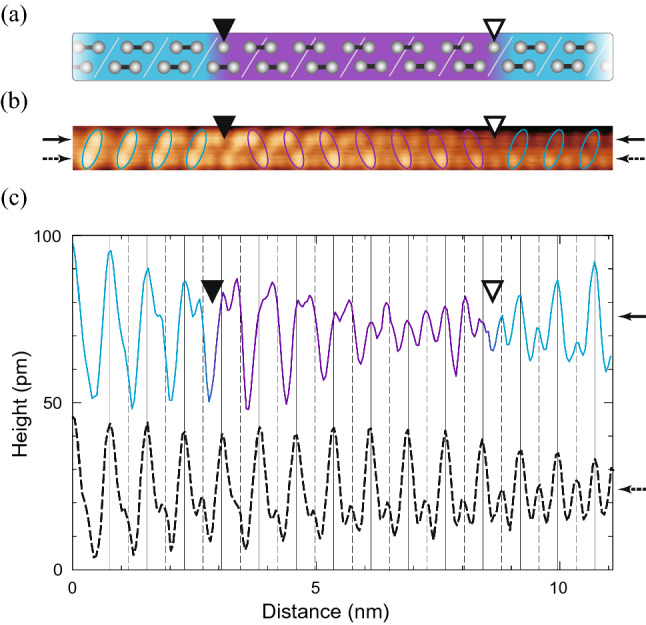
Experimental evidence of a pair creation of RC- and LC-solitons. (**a**) Atomic model of a pair of RC- and LC-solitons. (**b**) Scanning tunneling microscopy (STM) image showing a pair of RC- and LC-solitons. (**c**) STM line profiles taken from the STM image in (**b**). Solid and dashed profiles are taken at the upper and lower chains indicated by solid and dashed arrows in (**b**), respectively. The line profiles are high-pass filtered to get rid of slowly varying backgrounds. The distance between adjacent vertical solid and dashed lines is half the length of unit cell, $\frac{a_0}{2}$. In this figure, closed and open triangles indicate the kink positions of RC- and LC-solitons, respectively.

For topological algebraic operation using solitons, the manipulation of pair creation and pair annihilation of solitons is essential. Even though the recent experiment showed the switching between LC- and RC-solitons^21^, the pair creation and pair annihilation have not been experimentally reported mainly due to poor temporal resolution of experiments. However, the robustness of topological solitons and their PA dualities strongly suggest that the pair creation and pair annihilation should be observable with suitable experimental methods. Here, as an example, we present the experimental evidence of the RC- and LC-soliton pair generation in the prototypical In/Si(111) system using scanning tunneling microscopy (Fig. 4). Figure 4a shows the atomic model for pair created RC- and LC-solitons, where the upper chain has two separate kinks (indicated by closed and open triangles) while the lower chain does not have any kink. Figure 4b shows a scanning tunneling microscopy (STM) image of two chiral solitons corresponding to the same atomic model in Fig. 4a. To get more understanding, we take STM line profiles of both upper and lower chains from the STM image (Fig. 4c). In the upper line profile, each maximum in a unit cell is in phase with solid vertical lines at the left and right regions while each maximum is in phase with dashed vertical lines in the middle regions (or between two kinks). On the other hand, in the lower line profile, each maximum is﻿ always in phase with solid vertical lines. This observation strongly indicates that there are two soliton kinks on the upper chain and a single dimerized phase appears on the whole lower chain. This is consistent with the atomic model, showing the pair creation of $S_1^R$ and $S_4^L$.

## Conclusion

We have demonstrated that RC- and LC-solitons respect the PA duality and an AC-soliton does self PA duality using nonsymmorphic charge-conjugation operators. We found that “1st-fractionalized” solitons are “2nd-fractionalized” into two subsoliton states leading to the emergence of a quartet of subsolitons from the vacuum in the process of pair creation. As a result, we extracted the important information that supports not only the topological pair creation but also topological $\mathbb {Z}_4$ algebraic structures in the viewpoint of the topological operation. These theoretical concept and method can be easily generalized to other interesting topological systems such as 1D Rice-Mele^8^ and 2D Haldane models^35^ and the generalized model can be experimentally verified in various physical systems including atomic wires, cold atomic systems, and photonic crystals^36–38^. In the practical side, further study may focus on the fault-free manipulation of pair creation, pair annihilation, and topological operation among solitons in an appropriate time-scale. Such developments can in turn lead to developing topological quantum information technology and inspire researches seeking feasible and promising topological systems, which may lead to new practical applications such as solitronics.

## Methods

To study various properties of topological solitons, we used the tight-binding as well as Bloch and low-energy effective Hamiltonians of the double-Peierls-chain model^20,39^. The spatially resolved LDOS’s of three types of chiral solitons and their subsolitons were numerically obtained through the tight-binding calculation. The various PA dualities and equivalent relations under the three classes of nonsymmorphic operators were proved in the low-energy effective theory. By generalizing the Goldstone-Wilczek formula, the topological charges of each subsoliton states were calculated. To obtain the electrical charges of the solitons, we considered the neutralized systems where the Fermi-level is located at $E=0$. With the low-energy effective Hamiltonians, we analytically obtained all spectra of subsolitons. The fractionalized Berry curvatures for the primary and induced subsolitons of RC- and LC-solitons were calculated using the effective Hamiltonian of each chain. The fractionalized Berry curvatures for the bonding and antibonding subsoliton states of an AC-soliton were calculated using the effective bonding and antibonding Hamiltonians. To represent pseudospin vectors, we decomposed the wavefunction $\Psi (x)=[ A(x), B(x), C(x), D(x) ]$ on the four atoms in a unit cell into two spinors as $\Psi _{\text {chain 1}} = [ A(x), B(x)]$ and $\Psi _{\text {chain 2}} = [ C(x), D(x) ]$ and each decomposed spinor in the *i*-th chain $(i=1,2)$ is represented by a pseudospin vector $(d^{(i)}_x, d^{(i)}_y, d^{(i)}_z)$. All the details are provided in the Supplementary Information.

## Supplementary Information

Supplementary Information.

## References

[1] Dauxois T, Peyrard M (2006). Physics of Solitons.

[2] Jackiw R, Rebbi C (1976). Solitons with fermion number $$1/2$$. Phys. Rev. D.

[3] Su WP, Schrieffer JR, Heeger AJ (1979). Solitons in polyacetylene. Phys. Rev. Lett..

[4] Jackiw R, Schrieffer JR (1981). Solitons with fermion number $$\frac{1}{2}$$ in condensed matter and relativistic field theories. Nucl. Phys. B.

[5] Jackiw R, Semenoff G (1983). Continuum quantum field theory for a linearly conjugated diatomic polymer with fermion fractionization. Phys. Rev. Lett..

[6] Thouless DJ (1983). Quantization of particle transport. Phys. Rev. B.

[7] Heeger AJ, Kivelson S, Schrieffer JR, Su WP (1988). Solitons in conducting polymers. Rev. Mod. Phys..

[8] Rice MJ, Mele EJ (1982). Elementary excitations of a linearly conjugated diatomic polymer. Phys. Rev. Lett..

[9] Väyrynen JI, Ojanen T (2011). Chiral topological phases and fractional domain wall excitations in one-dimensional chains and wires. Phys. Rev. Lett..

[10] Efroni Y, Ilani S, Berg E (2017). Topological transitions and fractional charges induced by strain and a magnetic field in carbon nanotubes. Phys. Rev. Lett..

[11] Chen BG-G, Upadhyaya N, Vitelli V (2014). Nonlinear conduction via solitons in a topological mechanical insulator. Proc. Natl. Acad. Sci. USA.

[12] Atala M (2013). Direct measurement of the zak phase in topological bloch bands. Nat. Phys..

[13] Lohse M, Schweizer C, Zilberberg O, Aidelsburger M, Bloch I (2016). A Thouless quantum pump with ultracold bosonic atoms in an optical superlattice. Nat. Phys..

[14] Nakajima S (2016). Topological Thouless pumping of ultracold fermions. Nat. Phys..

[15] Zhou X-F (2017). Dynamically Manipulating Topological Physics and Edge Modes in a Single Degenerate Optical Cavity. Phys. Rev. Lett..

[16] St-Jean P (2017). Lasing in topological edge states of a one-dimensional lattice. Nat. Photon..

[17] Zhao H (2018). Topological hybrid silicon microlasers. Nat. Commun..

[18] Huda MN, Kezilebieke S, Ojanen T, Drost R, Liljeroth P (2020). Tuneable topological domain wall states in engineered atomic chains. NPJ Quantum Mater..

[19] Queraltó G (2020). Topological state engineering via supersymmetric transformations. Commun. Phys..

[20] Cheon S, Kim T-H, Lee S-H, Yeom HW (2015). Chiral solitons in a coupled double Peierls chain. Science.

[21] Kim T-H, Cheon S, Yeom HW (2017). Switching chiral solitons for algebraic operation of topological quaternary digits. Nat. Phys..

[22] Su WP, Schrieffer JR (1981). Fractionally charged excitations in charge-density-wave systems with commensurability 3. Phys. Rev. Lett..

[23] Li L, Xu Z, Chen S (2014). Topological phases of generalized Su–Schrieffer–Heeger models. Phys. Rev. B.

[24] Shiozaki K, Sato M, Gomi K (2015). $$Z_2$$ topology in nonsymmorphic crystalline insulators: Möbius twist in surface states. Phys. Rev. B.

[25] Zhao YX, Schnyder AP (2016). Nonsymmorphic symmetry-required band crossings in topological semimetals. Phys. Rev. B.

[26] Zhang S-L, Zhou Q (2017). Two-leg Su–Schrieffer–Heeger chain with glide reflection symmetry. Phys. Rev. A.

[27] Velasco CG, Paredes B (2017). Realizing and detecting a topological insulator in the AIII symmetry class. Phys. Rev. Lett..

[28] Xie D, Gou W, Xiao T, Gadway B, Yan B (2019). Topological characterizations of an extended Su–Schrieffer–Heeger model. NPJ Quantum Inf..

[29] Yeom HW (1999). Instability and charge density wave of metallic quantum chains on a silicon surface. Phys. Rev. Lett..

[30] Kim T-H, Yeom HW (2012). Topological solitons versus nonsolitonic phase defects in a quasi-one-dimensional charge-density wave. Phys. Rev. Lett..

[31] Elliott SR, Franz M (2015). Colloquium: Majorana fermions in nuclear, particle, and solid-state physics. Rev. Mod. Phys..

[32] Schnyder AP, Ryu S, Furusaki A, Ludwig AWW (2008). Classification of topological insulators and superconductors in three spatial dimensions. Phys. Rev. B.

[33] Goldstone J, Wilczek F (1981). Fractional quantum numbers on solitons. Phys. Rev. Lett..

[34] Qi X-L, Hughes TL, Zhang S-C (2008). Topological field theory of time-reversal invariant insulators. Phys. Rev. B.

[35] Haldane FDM (1988). Model for a quantum hall effect without landau levels: Condensed-matter realization of the “parity anomaly”. Phys. Rev. Lett..

[36] Snijders PC, Weitering HH (2010). Colloquium: Electronic instabilities in self-assembled atom wires. Rev. Mod. Phys..

[37] Ozawa T (2019). Topological photonics. Rev. Mod. Phys..

[38] Cooper NR, Dalibard J, Spielman IB (2019). Topological bands for ultracold atoms. Rev. Mod. Phys..

[39] Han S-H, Jeong S-G, Kim S-W, Kim T-H, Cheon S (2020). Topological features of ground states and topological solitons in generalized Su-Schrieffer-Heeger models using generalized time-reversal, particle-hole, and chiral symmetries. Phys. Rev. B.

