# Supplementary material for: Particle-antiparticle duality and fractionalization of topological chiral solitons
Source: Sci Rep. 2021 Jan 13;11:1013. doi: 10.1038/s41598-020-80085-8 (PMC7807002; doi:10.1038/s41598-020-80085-8)
Supplement: Supplementary file 1 — Supplementary Information. [file 41598_2020_80085_MOESM1_ESM.pdf]

Supplementary Information:
Particle-antiparticle duality and fractionalization of topological chiral solitons

Chang-geun Oh,[1,*] Sang-Hoon Han,[2,*] Seung-Gyo Jeong,[3,*] Tae-Hwan Kim,[3,†] and Sangmo Cheon[1,2,‡]

[1]*Research Institute for Natural Sciences, Hanyang University, Seoul 04763, Korea*
[2]*Department of Physics, Hanyang University, Seoul 04763, Korea*
[3]*Department of Physics, Pohang University of Science and Technology (POSTECH), Pohang 37673, Korea*
(Dated: December 4, 2020)

CONTENTS

S1. Double-Peierls-Chain Model 2
S1.1. Bloch Hamiltonian 2
S1.2. Low energy effective continuum Hamiltonian 2
S1.3. Tight-binding method and parameters 2
S1.4. Notations for twelve topological chiral solitons 2

S2. Symmetry Analysis for Topological Chiral Solitons 3
S2.1. Three classes of nonsymmorphic operators 3
S2.2. Transformations among topological chiral solitons 3
S2.3. PA duality between RC- and LC-solitons 4
S2.4. PA duality between AC-solitons 4
S2.5. Self PA duality and pseudo-self-charge-conjugate property of AC-solitons 5
S2.6. Self-conjugate property of SSH solitons 5

S3. Spectra and Fractional Fermion Numbers of Topological Chiral Solitons 5
S3.1. Spectra of RC-, LC-, and AC-solitons 5
S3.2. Fermion numbers of topological chiral solitons 6

S4. Berry Curvature and Fractionalized Berry Curvature 6

S5. Wavefunctions and Pseudospin Vectors of Topological Chiral Solitons 7
S5.1. Wavefunctions 7
S5.2. Pseudospin vectors 7
S5.3. PA duality between RC- and LC-solitons 8
S5.4. Self PA duality of AC-solitons 8
S5.5. Nonsymmorphic charge-conjugation operators in convention II 8

References 9

* These authors contributed equally to this work.
† taehwan@postech.ac.kr
‡ sangmocheon@hanyang.ac.kr

S1. DOUBLE-PEIERLS-CHAIN MODEL

S1.1. Bloch Hamiltonian

We consider the double-Peierls-chain model [1] which is composed of two identical SSH chains with interchain coupling (see inset of Fig. 1a in the main text). The Bloch Hamiltonian of this model is given by

$$\mathcal{H}(k_x, \Delta_1, \Delta_2) = \begin{pmatrix} \mathcal{H}_1 & \mathcal{H}_{12} \\ \mathcal{H}_{21} & \mathcal{H}_2 \end{pmatrix}, \tag{1}$$

where

$$\mathcal{H}_i = (2t_0 \cos(k_x a_0/2), -\Delta_i \sin(k_x a_0/2), 0) \cdot \boldsymbol{\sigma},$$
$$\mathcal{H}_{12} = \mathcal{H}_{21}^\dagger = \delta t_0 (e^{-ik_x a_0/4} 1_{2\times 2} + e^{ik_x a_0/4} \sigma_x).$$

Here, t_0 is the hopping integral between nearest atoms along the chain in the undimerized phase, a_0 is the lattice constant of the dimerized phases, δ is the interchain coupling strength, $\boldsymbol{\sigma} = (\sigma_x, \sigma_y, \sigma_z)$ are the Pauli matrices, and Δ_i is the energy-valued Peierls dimerization displacement of the i-th chain ($\Delta_i = 4\alpha u^{(i)}$). Here, $u^{(i)}$ is the atomic dimerization displacement for the i-th chain and α is the electron-phonon coupling coefficient. Thus, the strong and week hopping integrals in dimerized chains are given by $t_0 + \Delta_i/2$ and $t_0 - \Delta_i/2$, respectively.

S1.2. Low energy effective continuum Hamiltonian

The low energy effective continuum Hamiltonian is derived in the continuum limit near the $k_x = -\pi/a_0$:

$$\mathsf{H}(\Delta_1, \Delta_2) = \begin{pmatrix} \mathsf{H}_1 & \mathsf{H}_{12} \\ \mathsf{H}_{21} & \mathsf{H}_2 \end{pmatrix}, \tag{2}$$

with

$$\mathsf{H}_i = -iv_F \partial_x \sigma_x + \Delta_i(x) \sigma_y, \tag{3}$$

$$\mathsf{H}_{12} = \mathsf{H}_{21}^\dagger = \frac{t_0 \delta}{\sqrt{2}} \left[(1+i) 1_{2\times 2} + (1-i)\sigma_x\right], \tag{4}$$

where $v_F = t_0 a_0$ is the Fermi velocity. For simplicity, we set $\hbar = 1$. This continuum Hamiltonian allows the analytical form for the energy eigenvalue of the groundstate [$\Delta_i = \pm\Delta_0(\delta)$], which is given by

$$E_{k_x} = \pm\sqrt{A \pm B}, \tag{5}$$

where $A = v_F^2 k_x^2 + \Delta_0^2(\delta) + 2[t_0 \Delta_0(\delta)]^2$, $B = 2t_0 \Delta_0(\delta) \sqrt{2v_F^2 k_x^2 + \Delta_0^2(\delta)}$ and $\Delta_0(\delta)$ is the dimerization displacement of groundstates. Hence, there are four bands and the band gap is given by $E_{\text{gap}} = 2\sqrt{A - B}$.

S1.3. Tight-binding method and parameters

For the numerical simulation of topological soliton, we adopt the self-consistent tight-binding method [1]. Based on the real quasi-one dimensional system of In/Si(111) [1–4] and density functional theory calculations [5, 6], we choose the following parameter set that reproduces the experimentally measured band structure, soliton length, and soliton spectrum [1, 3, 4]: $a_0 = 7.70$ Å, $t_0 = 0.4$ eV, $K = 0.55$ eV/Å^2, $\alpha = 0.28$ eV/Å, and $\delta = 0.2$ is used in Fig. 1 of the main text. On the other hand, for Berry curvatures, pseudospin vectors, and wavefunctions in Figs. 3 and S2, we choose $\delta = 0.1$ for convenience. The energy-valued dimerization displacement is self-consistently determined: $\Delta_0 = 0.122$ eV for $\delta = 0.2$ and $\Delta_0 = 0.134$ eV for $\delta = 0.1$.

S1.4. Notations for twelve topological chiral solitons

In the double-Peierls-chain model, the twelve topological chiral solitons shown in the order-parameter space of Fig. 1b in the main text are listed in Table S1.

Soliton type	Notation	Phase shift	$\Delta_1(x)$	$\Delta_2(x)$
RC	S_1^R	AA $\rightarrow$ BA	$+\Delta_0 \rightarrow -\Delta_0$	$+\Delta_0$
	S_2^R	BA $\rightarrow$ BB	$-\Delta_0$	$+\Delta_0 \rightarrow -\Delta_0$
	S_3^R	BB $\rightarrow$ AB	$-\Delta_0 \rightarrow +\Delta_0$	$-\Delta_0$
	S_4^R	AB $\rightarrow$ AA	$+\Delta_0$	$-\Delta_0 \rightarrow +\Delta_0$
LC	S_1^L	BB $\rightarrow$ BA	$-\Delta_0$	$-\Delta_0 \rightarrow +\Delta_0$
	S_2^L	AB $\rightarrow$ BB	$+\Delta_0 \rightarrow -\Delta_0$	$-\Delta_0$
	S_3^L	AA $\rightarrow$ AB	$+\Delta_0$	$+\Delta_0 \rightarrow -\Delta_0$
	S_4^L	BA $\rightarrow$ AA	$-\Delta_0 \rightarrow +\Delta_0$	$+\Delta_0$
AC	S_1^A	AA $\rightarrow$ BB	$+\Delta_0 \rightarrow -\Delta_0$	$+\Delta_0 \rightarrow -\Delta_0$
	S_2^A	BA $\rightarrow$ AB	$-\Delta_0 \rightarrow +\Delta_0$	$+\Delta_0 \rightarrow -\Delta_0$
	S_3^A	BB $\rightarrow$ AA	$-\Delta_0 \rightarrow +\Delta_0$	$-\Delta_0 \rightarrow +\Delta_0$
	S_4^A	AB $\rightarrow$ BA	$+\Delta_0 \rightarrow -\Delta_0$	$-\Delta_0 \rightarrow +\Delta_0$

Table S1. Notations for twelve topological chiral solitons and their phase shifts in the order parameter space. For the soliton profiles, $\Delta_i(x)$ at $x = \pm\infty$ are represented. Δ_0 is the energy-valued dimerization displacement of groundstates.

S2. SYMMETRY ANALYSIS FOR TOPOLOGICAL CHIRAL SOLITONS

In this section, we explicitly prove transformation properties among topological chiral solitons in low energy effective theory using three classes of nonsymmorphic operators.

S2.1. Three classes of nonsymmorphic operators

The first one is the glide reflection operator $\hat{G}_y = \{M_y| -\frac{a_0}{4}\}$ which flips the atoms in the upper and lower chains with respect to the horizontal line between two chains (or mirror reflection operation M_y as shown in Fig. S1) followed by a fractional translation $-\frac{a_0}{4}$ along the chain direction. The second class operator is the nonsymmorphic charge-conjugation operator $\hat{\mathcal{C}}^{(i)}_{\mathrm{RL}} = \hat{G}^{(i)}\hat{\mathcal{C}}$ that gives the PA duality between RC- and LC-solitons, where $\hat{G}^{(i)} \equiv \{E|(-1)^{i-1}\frac{a_0}{2}\}^{(i)}$ is a fractional translation operator along the i-th chain only. The third class operator is the nonsymmorphic charge-conjugation operator $\hat{\mathcal{C}}^{(i)}_{\mathrm{AC}}$ that gives the PA dualities among AC-solitons. Their representations, cyclic properties, and roles are explicitly listed in Table S2.

S2.2. Transformations among topological chiral solitons

In this subsection, we explicitly prove the three relations among topological chiral solitons under the three classes of the nonsymmorphic operators using the low energy effective Hamiltonians. The results are summarized in Table S3.

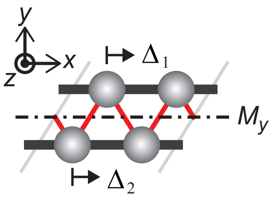

Fig. S1. Mirror reflection operation M_y with respect to the horizontal xz plane (indicated by a dashed line).

Operator	Bloch	Continuum	Cyclic	Role
$\hat{G}_y \equiv \{M_y \vert -\frac{a_0}{4}\}$	$e^{i\frac{ka_0}{4}}\begin{pmatrix}0 & \sigma_x\\ \mathbf{1} & 0\end{pmatrix}$	$e^{-i\frac{\pi}{4}}\begin{pmatrix}0 & \sigma_x\\ \mathbf{1} & 0\end{pmatrix}$	$(\hat{G}_y)^4 = 1$	Equivalence
$\hat{\mathcal{C}}^{(1)}_{\text{RL}} \equiv \hat{G}^{(1)}\hat{\mathcal{C}}$	$\begin{pmatrix}-ie^{-i\frac{ka_0}{2}}\sigma_y & 0\\ 0 & \sigma_z\end{pmatrix}\hat{K}\otimes(k\to -k)$	$i\begin{pmatrix}\sigma_x & 0\\ 0 & -\mathbf{1}\end{pmatrix}\hat{K}$	$(\hat{\mathcal{C}}^{(1)}_{\text{RL}})^2 = 1$	PA duality
$\hat{\mathcal{C}}^{(2)}_{\text{RL}} \equiv \hat{G}^{(2)}\hat{\mathcal{C}}$	$\begin{pmatrix}\sigma_z & 0\\ 0 & -ie^{+i\frac{ka_0}{2}}\sigma_y\end{pmatrix}\hat{K}\otimes(k\to -k)$	$\begin{pmatrix}\mathbf{1} & 0\\ 0 & -\sigma_x\end{pmatrix}\hat{K}$	$(\hat{\mathcal{C}}^{(2)}_{\text{RL}})^2 = 1$	PA duality
$\hat{\mathcal{C}}^{(1)}_{\text{AC}} \equiv \hat{\mathcal{C}}^{(1)}_{\text{RL}}\hat{G}_y$	$e^{i\frac{ka_0}{4}}\begin{pmatrix}0 & -e^{-i\frac{ka_0}{2}}\sigma_z\\ \sigma_z & 0\end{pmatrix}\hat{K}\otimes(k\to -k)$	$ie^{i\frac{\pi}{4}}\begin{pmatrix}0 & \mathbf{1}\\ -\mathbf{1} & 0\end{pmatrix}\hat{K}$	$(\hat{\mathcal{C}}^{(1)}_{\text{AC}})^2 = -1$	PA duality
$\hat{\mathcal{C}}^{(2)}_{\text{AC}} \equiv \hat{G}_y^{-1}\hat{\mathcal{C}}^{(2)}_{\text{RL}}$	$-ie^{i\frac{ka_0}{4}}\begin{pmatrix}0 & e^{i\frac{ka_0}{2}}\sigma_y\\ \sigma_y & 0\end{pmatrix}\hat{K}\otimes(k\to -k)$	$ie^{i\frac{\pi}{4}}\begin{pmatrix}0 & -\sigma_x\\ \sigma_x & 0\end{pmatrix}\hat{K}$	$(\hat{\mathcal{C}}^{(2)}_{\text{AC}})^2 = -1$	PA duality

Table S2. Three types of nonsymmorphic symmetry operators in the Bloch basis and low energy continuum theory. Here, the representations of the charge conjugation operator $\hat{\mathcal{C}}$ in the Bloch basis and low energy continuum theory are given by $\begin{pmatrix}\sigma_z & 0\\ 0 & \sigma_z\end{pmatrix}\hat{K}$ and $\begin{pmatrix}\mathbf{1} & 0\\ 0 & -i\mathbf{1}\end{pmatrix}\hat{K}$, respectively. $\hat{K}$ is the complex conjugate operator.

Consider a soliton solution described by a wavefunction $\Psi(x)$ and dimerization profiles $[\Delta_1(x), \Delta_2(x)]$ with an energy eigenvalue E that satisfy the following eigenvalue equation:

$$\mathsf{H}(\Delta_1(x), \Delta_2(x))\Psi(x) = E\Psi(x). \tag{6}$$

Then, the transformed soliton under an operator $\hat{O}$ is also a soliton solution which is described by $\Psi'(x) \equiv \hat{O}\Psi(x)$ and $[\Delta_1'(x), \Delta_2'(x)]$ with $E' = \eta E$ ($\eta = \pm 1$) because the low energy continuum Hamiltonian H transforms under $\hat{O}$ as

$$\hat{O}\mathsf{H}(\Delta_1(x), \Delta_2(x))\hat{O}^{-1} = \eta\mathsf{H}(\Delta_1'(x), \Delta_2'(x)). \tag{7}$$

Depending on the dimerization profiles $[\Delta_1'(x), \Delta_2'(x)]$, the transformed soliton corresponds to either RC-, LC-, or AC-soliton.

For example, under the operator $\hat{G}_y$, the transformed soliton has $[\Delta_1'(x), \Delta_2'(x)] = [-\Delta_2(x), \Delta_1(x)]$, and $E' = E$. Thus, the solitons with the same chirality are physically equivalent and have the same energy eigenvalues. Symbolically, we find that

$$S_1^k = \hat{G}_y S_4^k = \hat{G}_y^2 S_3^k = \hat{G}_y^3 S_2^k = \hat{G}_y^4 S_1^k, \tag{8}$$

where k is either R, L or A.

S2.3. PA duality between RC- and LC-solitons

Let us consider the PA-duality between RC- and LC-solitons via the nonsymmorphic charge-conjugation operator $\hat{\mathcal{C}}^{(i)}_{\text{RL}}$ illustrated in Fig. 2b of the main text. Under $\hat{\mathcal{C}}^{(i)}_{\text{RL}}$, an RC-soliton having the dimerization profile (Δ_1, Δ_2) and an energy eigenvalue E transforms into an LC-soliton having $[\Delta_1'(x), \Delta_2'(x)] = [(-1)^i\Delta_1, (-1)^{i+1}\Delta_2]$ and the opposite energy eigenvalue $-E$. For instance, under $\hat{\mathcal{C}}^{(1)}_{\text{RL}}$, S_1^R is transformed into S_4^L exchanging the energy spectra, leading to PA duality between RC- and LC-solitons. Moreover, the RC- and LC-soliton wavefunctions $[\Psi_{\text{RC}}(x)$ and $\Psi_{\text{LC}}(x)]$ also satisfy the PA duality:

$$\Psi_{\text{LC}}(x) = \hat{\mathcal{C}}^{(1)}_{\text{RL}}\Psi_{\text{RC}}(x) = i\begin{pmatrix}\sigma_x & 0\\ 0 & -\mathbf{1}\end{pmatrix}\Psi^*_{\text{RC}}(x). \tag{9}$$

In the view point of quantum field theory, the wavefunctions can be promoted to the field creation and annihilation operators. Thus, Eq. (9) indicates the PA duality between an RC- and LC-soliton pair in the quantum level.

S2.4. PA duality between AC-solitons

Similarly, one can derive the PA duality between two AC-solitons shown in Fig. 2c of the main text. See the rows for the PA duality of $\hat{\mathcal{C}}^{(i)}_{\text{AC}}$ in Table S3.

Operator	E'	$\Delta'_1(x)$	$\Delta'_2(x)$	Soliton relation	Role
$\hat{G}_y$	$+E$	$-\Delta_2(x)$	$+\Delta_1(x)$	$\hat{G}_y S_i^k = S_{i+1}^k$ $(i=1,2,3,4)$	equivalence
$\hat{\mathcal{C}}_{\rm RL}^{(1)}$	$-E$	$-\Delta_1(x)$	$+\Delta_2(x)$	$\hat{\mathcal{C}}_{\rm RL}^{(1)} S_i^{R(L)} = S_{5-i}^{L(R)}$ $(i=1,2,3,4)$	PA duality
$\hat{\mathcal{C}}_{\rm RL}^{(2)}$	$-E$	$+\Delta_1(x)$	$-\Delta_2(x)$	$\hat{\mathcal{C}}_{\rm RL}^{(2)} S_i^{R(L)} = S_{i+(-1)^{i+1}}^{L(R)}$ $(i=1,2,3,4)$	PA duality
$\hat{\mathcal{C}}_{\rm AC}^{(1)}$	$-E$	$+\Delta_2(x)$	$+\Delta_1(x)$	$\hat{\mathcal{C}}_{\rm AC}^{(1)} S_i^A = S_i^A$ $(i=1,3)$	self PA duality
				$\hat{\mathcal{C}}_{\rm AC}^{(1)} S_i^A = S_{6-i}^A$ $(i=2,4)$	PA duality
$\hat{\mathcal{C}}_{\rm AC}^{(2)}$	$-E$	$-\Delta_2(x)$	$-\Delta_1(x)$	$\hat{\mathcal{C}}_{\rm AC}^{(2)} S_i^A = S_i^A$ $(i=2,4)$	self PA duality
				$\hat{\mathcal{C}}_{\rm AC}^{(2)} S_i^A = S_{4-i}^A$ $(i=1,3)$	PA duality

Table S3. Transformation properties of topological chiral solitons under three classes of nonsymmorphic operators. Before a transformation, a soliton has an energy eigenvalue E and dimerization profiles $[\Delta_1(x), \Delta_2(x)]$, while the transformed soliton has the energy eigenvalue E' and dimerization profiles $[\Delta'_1(x), \Delta'_2(x)]$. In the column 'soliton relation', $S_5^k = S_1^k$ and $k = R, L, A$ are used.

S2.5. Self PA duality and pseudo-self-charge-conjugate property of AC-solitons

Let us consider the self PA duality of each AC-soliton via the nonsymmorphic charge-conjugation operator $\hat{\mathcal{C}}_{\rm AC}^{(i)}$ illustrated in Fig. 2d of the main text. Under $\hat{\mathcal{C}}_{\rm AC}^{(i)}$, an AC-soliton having the dimerization profile (Δ_1, Δ_2) and an energy eigenvalue E transforms into an AC-soliton having $[\Delta'_1(x), \Delta'_2(x)] = [(-1)^{i+1}\Delta_2(x), (-1)^{i+1}\Delta_1(x)]$ and the opposite energy eigenvalue $-E$. Thus, an AC-soliton can transform into itself with the opposite eigenvalue $-E$ (see the rows for the self PA duality of $\hat{\mathcal{C}}_{\rm AC}^{(i)}$ in Table S3), which implies the AC-soliton has a particle-hole symmetric spectra $\pm E$. For instance, the bonding and antibonding energy spectra of an AC-soliton (S_1^A) are exchanged leading to the self PA duality as shown in Fig. 1f of the main text (dashed green arrow). In addition, the bonding and antibonding wavefunctions of the AC-soliton, $\Psi_{\rm B}(x)$ and $\Psi_{\rm AB}(x)$, satisfy the PA relation:

$$\Psi_{\rm B} \propto \hat{\mathcal{C}}_{\rm AC}^{(1)} \Psi_{\rm AB} = i e^{i\frac{\pi}{4}} \begin{pmatrix} 0 & \mathbf{1} \\ -\mathbf{1} & 0 \end{pmatrix} \Psi_{\rm AB}^*. \tag{10}$$

Since $(\hat{\mathcal{C}}_{\rm AC}^{(i)})^2 = -1$, the bonding and antibonding states of each AC-soliton are naturally orthogonal like a time-reversal Kramer pair. Moreover, the combined bonding and antibonding subsoliton wavefunction $\Psi_{\rm AC} = (\Psi_{\rm AB}, \Psi_{\rm B})^{\rm T}$ is pseudo-self-charge-conjugate:

$$\begin{pmatrix} \Psi_{\rm AB} \\ \Psi_{\rm B} \end{pmatrix} = U \begin{pmatrix} \Psi_{\rm AB} \\ \Psi_{\rm B} \end{pmatrix}^*, \tag{11}$$

where U is an 8×8 unitary matrix.

S2.6. Self-conjugate property of SSH solitons

For the SSH model, the soliton S solution is given by $\Psi_S(x) = N_0(\mathrm{sech}(x/\xi), 0)^{\rm T}$ with the energy eigenvalue $E = 0$, where $\xi = \hbar v_F/\Delta_0$ and $N_0 = 1/\sqrt{2\xi}$. Similarly, the antisoliton $\bar{S}$ solution is given by $\Psi_{\bar{S}}(x) = N_0[0, \mathrm{sech}(x/\xi)]^{\rm T}$ with the energy eigenvalue $E = 0$. Thus, the SSH solitons satisfy the self-conjugate relation under the charge conjugate operation [7]. Mathematically, $\Psi_S(x) = \Psi_S^*(x)$ and $\Psi_{\bar{S}}(x) = \Psi_{\bar{S}}^*(x)$.

S3. SPECTRA AND FRACTIONAL FERMION NUMBERS OF TOPOLOGICAL CHIRAL SOLITONS

S3.1. Spectra of RC-, LC-, and AC-solitons

For convenience, we take a unitary transformation $\mathsf{H} \to U \mathsf{H} U^{-1}$, where $U = \begin{pmatrix} \mathbf{1}_{2\times 2} & 0 \\ 0 & e^{i\frac{\pi}{4}} \mathbf{1}_{2\times 2} \end{pmatrix}$. The phase factor $e^{i\frac{\pi}{4}}$ comes from a quarter translation of the second chain with respect to the first chain. Then, the transformed Hamiltonian is given

by

$$\mathsf{H}_i = -iv_F\partial_x\sigma_x + \Delta_i(x)\sigma_y, \tag{12}$$

$$\mathsf{H}_{12} = \mathsf{H}_{21}^\dagger = t_0\delta\left(\mathbf{1}_{2\times 2} - i\sigma_x\right). \tag{13}$$

The eigenvalue equation $\mathsf{H}\Psi = E\Psi$ for the soliton wavefunction $\Psi = (\mathsf{U},\mathsf{V})^{\mathrm{T}}$ is given by

$$\mathsf{H}_1\mathsf{U} + \mathsf{H}_{12}\mathsf{V} = E\mathsf{U}, \tag{14}$$

$$\mathsf{H}_2\mathsf{V} + \mathsf{H}_{21}\mathsf{U} = E\mathsf{V}, \tag{15}$$

where U and V are two-component spinor wavefunctions for the upper and lower chains, respectively. These equations are decoupled into two effective Hamiltonians for each chain. Using the slowly-varying soliton field approximation in space, the two effective Hamiltonians can be written as

$$H_{\text{eff}}^{(1)}\mathsf{U} = \left[-i\partial_x\sigma_x + \Delta_1(x)\sigma_y + \frac{2t_0^2\delta^2}{E^2 + {\partial_x}^2 - (\Delta_2)^2}\left(E - i\partial_x\sigma_x + \Delta_2\sigma_z\right)\right]\mathsf{U} = E\mathsf{U}, \tag{16}$$

$$H_{\text{eff}}^{(2)}\mathsf{V} = \left[-i\partial_x\sigma_x + \Delta_2(x)\sigma_y + \frac{2t_0^2\delta^2}{E^2 + {\partial_x}^2 - (\Delta_1)^2}\left(E - i\partial_x\sigma_x - \Delta_1\sigma_z\right)\right]\mathsf{V} = E\mathsf{V}, \tag{17}$$

where we set $v_F = 1$ for simplicity. Using these effective Hamiltonians, we obtain the energy eigenvalues of RC- and LC-solitons in a similar way of the Rice-Mele method [8]. For an RC-soliton, the primary and induced subsoliton energy spectra are given by

$$E_{\text{P}}^{\text{RC}} = -\frac{2t_0^2\delta^2}{\Delta_0},\;\; E_{\text{I}}^{\text{RC}} = -\Delta_0 + t_0\delta - \frac{t_0^2\delta^2}{4\Delta_0}$$

in the leading order. For an LC-soliton, the primary and induced subsoliton energy spectra are given by $E_{\text{P}}^{\text{LC}} = -E_{\text{P}}^{\text{RC}}$ and $E_{\text{I}}^{\text{LC}} = -E_{\text{I}}^{\text{RC}}$, which explicitly supports the PA duality between RC- and LC-solitons. In a similar way, we obtain bonding and antibonding subsoliton spectra of an AC-soliton considering the hybridized wavefunction $U \pm V$. The spectra are given by $E_{\text{B}}^{\text{AC}} = -E_{\text{AB}}^{\text{AC}} = -t_0\delta_0$, which supports the self PA duality of an AC-soliton.

S3.2. Fermion numbers of topological chiral solitons

For an effective Dirac Hamiltonian $H_{\text{eff}}(x) = -i\partial_x\sigma_x + m_x(x)\sigma_y + m_z(x)\sigma_z$, the fermion number of a soliton is given by $N = \left[\frac{1}{2\pi}\tan^{-1}\frac{m_z(x)}{m_x(x)}\right]_{x=-\infty}^{x=\infty}$, where $[m_x(x), m_z(x)]$ are scalar fields that support a soliton [9]. Now, using the effective Hamiltonian for each chain, we calculate the fermion numbers of RC- and LC-solitons. If a soliton kink resides in chain 1, for instance, the fermion numbers of the primary and induced subsolitons are given by $N_{\text{P}}^{\text{RC/LC}} = -\frac{1}{2} + \frac{1}{2}N_1^{(1)} + \frac{1}{2}N_2^{(1)}$ and $N_{\text{I}}^{\text{RC/LC}} = \frac{1}{2}N_1^{(2)} + \frac{1}{2}N_2^{(2)}$, where $N_n^{(i)}$ is the fermion number from i-th chain and n-th band. Here, $n = 1$ and $n = 2$ indicate the filled lowest and next lowest bands, respectively. Because the effective Hamiltonian is reduced to 2×2 Hamiltonian comparing to the original 4×4 Hamiltonian, the normalization factor $1/2$ is needed for the total fermion number conservation. Similarly, for an AC-soliton, the fermion numbers of bonding and antibonding subsoliton states are obtained as $N_{\text{B}} = N_{\text{AB}} = -1/2$.

S4. BERRY CURVATURE AND FRACTIONALIZED BERRY CURVATURE

We calculate topological charges of a soliton and its subsolitons using Berry curvature and fractionalized Berry curvatures under an adiabatic evolution, respectively [1, 10, 11]. The adiabatic evolution is generated by transporting a topological soliton slowly, which is represented by the time-dependent phase-space Hamiltonian $\mathsf{H}[k_x, t]$ with time-varying displacement fields $[\Delta_1(t), \Delta_2(t)]$. For example, transporting an RC-soliton S_1^R generates an adiabatic evolution of $AA \rightarrow BA$. Then, the total topological charge of a soliton is given by $Q = eC_{\text{sol}}$ where C_{sol} is the phase-space partial Chern number obtained under the corresponding adiabatic evolution. The phase-space partial Chern number for multibands from the initial time t_i to the final time t_f (which corresponds to a soliton) is defined as

$$C_{\text{sol}} = \frac{i}{2\pi}\sum_{n=\text{occ}}\int_{\text{BZ}} dk_x \int_{t_i}^{t_f} dt\,\Omega_n, \tag{18}$$

where the summation is done over the occupied bands and $\Omega_n = \langle\partial_{k_x}u_n\,|\,\partial_t u_n\rangle - \langle\partial_t u_n\,|\,\partial_{k_x}u_n\rangle$ is the phase-space Berry curvature for n-th band. Here, $|u_n\rangle$ is a normalized eigenvector of n-th band for the Hamiltonian $\mathsf{H}[k_x, t]$.

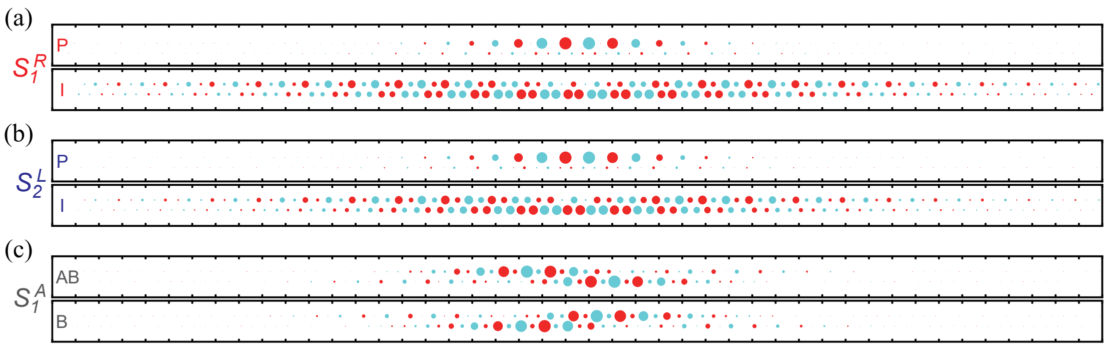

Fig. S2. **(a)–(c)** Representative wavefunctions for (a) RC-, (b) LC-, and (c) AC-subsolitons along the chain in the real space. The amplitude and phase of the wavefunctions are represented by the size of circles and colors. Red and cyan colors indicate 0 and π phases, respectively. In (a) and (b), 'P' and 'I' stand for primary and induced subsolitons, respectively. In (c), 'B' and 'AB' denote boding and antibonding AC-subsoliton states, respectively.

The Chern numbers for RC- and LC-solitons can be decomposed into fractionalized Chern numbers of primary and induced subsoliton states; $C_{\text{sol}} = C_{\text{P}} + C_{\text{I}}$ where

$$C_{\text{P/I}} = \frac{i}{2\pi} \sum_{n=\text{occ}} \int_{\text{BZ}} dk_x \int_{t_i}^{t_f} dt\, \Omega_{\text{P/I}}. \tag{19}$$

Here, Ω_{P} and Ω_{I} are the fractionalized Berry curvatures for the primary and induced subsoliton states, which are given by $\Omega_{\text{P}} = \Omega_1^{(1)} + \Omega_2^{(1)}$, $\Omega_{\text{I}} = \Omega_1^{(2)} + \Omega_2^{(2)}$. $\Omega_n^{(i)}$ is the fractionalized Berry curvature for n-th band using an effective Hamiltonian of the i-th chain, which is given by

$$\Omega_n^{(i)} = \frac{1}{2}\left(\left\langle \partial_{k_x} u_n^{(i)} \middle| \partial_t u_n^{(i)} \right\rangle - \left\langle \partial_t u_n^{(i)} \middle| \partial_{k_x} u_n^{(i)} \right\rangle\right).$$

Here, $\left| u_n^{(i)} \right\rangle$ is a normalized eigenvector of n-th band for the effective Hamiltonian of the i-th chain $\mathsf{H}_{\text{eff}}^{(i)}[k_x, t]$.

Because the bonding and antibonding subsoliton states are generated by the hybridization of two SSH solitons via the inter-chain coupling, the topological charges also come from effective bonding and antibonding Hamiltonians. Hence, the fractionalized Berry curvatures for the bonding and antibonding subsoliton states of an AC-soliton are given by $\Omega_{\text{B}} = \Omega_1^{(\text{B})} + \Omega_2^{(\text{B})}$, $\Omega_{\text{AB}} = \Omega_1^{(\text{AB})} + \Omega_2^{(\text{AB})}$, where $\Omega_n^{(\text{B/AB})}$ is the fractionalized Berry curvature for the n-th band of the effective bonding/antibonding Hamiltonian.

In Fig. 3g–i of the main text, the numerically calculated (fractionalized) Berry curvatures are shown in the (k_x, k_y) space, where the time t is replaced by the k_y using the dimensional extension [1, 10].

S5. WAVEFUNCTIONS AND PSEUDOSPIN VECTORS OF TOPOLOGICAL CHIRAL SOLITONS

S5.1. Wavefunctions

Figure S2a, b shows the numerically calculated wavefunctions of primary and induced subsolitons for RC- and LC-solitons, respectively. The primary subsoliton states predominantly reside in the main chain where the soliton kink is positioned while the induced subsoliton states reside in the other chain. Figure S2c shows that the bonding and antibonding states of an AC-soliton equally reside in both chains due to the self PA duality. The relative phase between wavefunctions at upper and lower chains distinguishes bonding and antibonding states.

S5.2. Pseudospin vectors

Various PA dualities among the subsolitons can be easily visualized by the pseudospin vectors along each chain as discussed in the main text. The wavefunctions on the four atoms in the unit-cell are decomposed into two spinors (one spinor to each chain), and each spinor is represented by a pseudospin vector $(d_x^{(i)}, d_y^{(i)}, d_z^{(i)})$ along the i-th chain (Fig. 3j–l of the main text).

Thus, the pseudospin vector corresponds to the spinor wavefunction

$$|u\rangle = (e^{-i\phi}\sin\frac{\theta}{2}, -\cos\frac{\theta}{2})^{\mathrm{T}}, \tag{20}$$

where θ and ϕ are polar and azimuthal angles of the pseudospin vector, respectively.

S5.3. PA duality between RC- and LC-solitons

We now explicitly prove that the PA duality between wavefunctions of RC- and LC-subsolitons using the nonsymmorphic charge-conjugation operators $\hat{\mathcal{C}}^{(i)}_{\mathrm{RL}}$. Since the RC- and LC-solitons (S_1^R and S_2^L) satisfy the PA duality of $S_2^L = \hat{\mathcal{C}}^{(2)}_{\mathrm{RL}} S_1^R$, each fractionalized wavefunction of RC- and LC-solitons [$\Psi^{\mathrm{RC}}_{\mathrm{P/I}}(x)$ and $\Psi^{\mathrm{LC}}_{\mathrm{P/I}}(x)$] also satisfies the PA duality:

$$\Psi^{\mathrm{LC}}_{\mathrm{P/I}}(x) \propto \hat{\mathcal{C}}^{(2)}_{\mathrm{RL}}\Psi^{\mathrm{RC}}_{\mathrm{P/I}}(x) = \begin{pmatrix} \sigma_z & 0 \\ 0 & -\sigma_x \end{pmatrix} \left[\Psi^{\mathrm{RC}}_{\mathrm{P/I}}(x)\right]^*,$$

where the convention II for the Bloch wavefunction is used (see Table S4). Then, the direction vector of a pseudospin in chain 1 transforms as $(d_x^{(1)}, d_y^{(1)}, d_z^{(1)}) \to (-d_x^{(1)}, d_y^{(1)}, d_z^{(1)})$. Similarly, for chain 2, $(d_x^{(2)}, d_y^{(2)}, d_z^{(2)}) \to (d_x^{(2)}, d_y^{(2)}, -d_z^{(2)})$. Thus, the pseudospin vectors for S_1^R and S_2^L rotate oppositely in the xz plane, which is consistent with the numerical results shown in Fig. 3j, k of the main text.

S5.4. Self PA duality of AC-solitons

Similarly, bonding and antibonding wavefunctions of an AC-soliton satisfy the self PA duality. For instance, from the relation of $S_1^A = \hat{\mathcal{C}}^{(1)}_{\mathrm{AC}} S_1^A$, the wavefunctions of the bonding and antibonding states [$\Psi_{\mathrm{B}}(x)$ and $\Psi_{\mathrm{AB}}(x)$] are related as

$$\Psi_{\mathrm{AB}}(x) \propto \hat{\mathcal{C}}^{(1)}_{\mathrm{AC}}\Psi_{\mathrm{B}}(x) = \begin{pmatrix} 0 & -\sigma_z \\ \sigma_z & 0 \end{pmatrix} \left[\Psi_{\mathrm{B}}(x)\right]^*,$$

where the convention II for the Bloch wavefunction is used (see Table S4). Then, the pseudospin vectors are transformed as $(d_x^{(1)}, d_y^{(1)}, d_z^{(1)}) \to (-d_x^{(2)}, d_y^{(2)}, d_z^{(2)})$ and $(d_x^{(2)}, d_y^{(2)}, d_z^{(2)}) \to (-d_x^{(1)}, d_y^{(1)}, d_z^{(1)})$, which explains the numerically obtained pseudospin profiles of the bonding and antibonding soliton states in Fig. 3l of the main text.

S5.5. Nonsymmorphic charge-conjugation operators in convention II

For the Bloch Hamiltonian and the symmetry analysis in Sec. S1 and S2, we used the convention I [12]. In convention I, the Bloch wavefunction has the exponential factor for each atom:

$$\psi_k(x) = \sum_{n=1}^{N}\sum_{j=1}^{4} e^{ikR_n}\left[C_{n,j}e^{ikr_j}\phi^j(x - R_n - r_j)\right],$$

Operator	Continuum	Role
$\hat{\mathcal{C}}^{(1)}_{\mathrm{RL}}$	$i\begin{pmatrix} \sigma_x & 0 \\ 0 & \sigma_z \end{pmatrix}\hat{K}$	PA duality
$\hat{\mathcal{C}}^{(2)}_{\mathrm{RL}}$	$\begin{pmatrix} \sigma_z & 0 \\ 0 & -\sigma_x \end{pmatrix}\hat{K}$	PA duality
$\hat{\mathcal{C}}^{(1)}_{\mathrm{AC}}$	$\begin{pmatrix} 0 & -\sigma_z \\ \sigma_z & 0 \end{pmatrix}\hat{K}$	PA duality
$\hat{\mathcal{C}}^{(2)}_{\mathrm{AC}}$	$\begin{pmatrix} 0 & -\sigma_x \\ \sigma_x & 0 \end{pmatrix}\hat{K}$	PA duality

Table S4. Representations of the nonsymmorphic charge-conjugation operators for the Bloch basis and low energy continuum theory in the convention II.

where $\phi^j(x - R_n - r_j)$ is the basis orbital of j-th atom in the n-th cell, R_n is the position vector of the n-th cell's center, r_j is the relative position vector of the j-th atom in the unit-cell, and $C_{n,j}$ is the coefficient.

For the wavefunction and pseudospin analysis in Sec. S5, we used the convention II where the exponential factor for each atom is not included:

$$\psi_k(x) = \sum_{n=1}^{N} \sum_{j=1}^{4} e^{ikR_n} \left[\tilde{C}_{n,j} \phi^j(x - R_n - r_j) \right],$$

where $\tilde{C}_{n,j} = e^{ikr_j} C_{n,j}$. The representations for nonsymmorphic chiral operators in convention II are summarized in Table S4.

---

[1] Cheon, S., Kim, T.-H., Lee, S.-H. & Yeom, H. W. Chiral solitons in a coupled double Peierls chain. *Science* **350**, 182–185 (2015).

[2] Yeom, H. W. *et al.* Instability and Charge Density Wave of Metallic Quantum Chains on a Silicon Surface. *Phys. Rev. Lett.* **82**, 4898–4901 (1999).

[3] Kim, T.-H. & Yeom, H. W. Topological Solitons versus Nonsolitonic Phase Defects in a Quasi-One-Dimensional Charge-Density Wave. *Phys. Rev. Lett.* **109**, 246802 (2012).

[4] Kim, T.-H., Cheon, S. & Yeom, H. W. Switching chiral solitons for algebraic operation of topological quaternary digits. *Nat. Phys.* **13**, 444–447 (2017).

[5] González, C., Ortega, J. & Flores, F. Metal–insulator transition in one-dimensional In-chains on Si(111): combination of a soft shear distortion and a double-band Peierls instability. *New J. Phys.* **7**, 100 (2005).

[6] Jeckelmann, E., Sanna, S., Schmidt, W. G., Speiser, E. & Esser, N. Grand canonical Peierls transition in In/Si(111). *Phys. Rev. B* **93**, 241407 (2016).

[7] Jackiw, R. & Schrieffer, J. R. Solitons with fermion number $\frac{1}{2}$ in condensed matter and relativistic field theories. *Nucl. Phys. B* **190**, 253–265 (1981).

[8] Rice, M. J. & Mele, E. J. Elementary Excitations of a Linearly Conjugated Diatomic Polymer. *Phys. Rev. Lett.* **49**, 1455–1459 (1982).

[9] Goldstone, J. & Wilczek, F. Fractional Quantum Numbers on Solitons. *Phys. Rev. Lett.* **47**, 986–989 (1981).

[10] Qi, X.-L., Hughes, T. L. & Zhang, S.-C. Topological field theory of time-reversal invariant insulators. *Phys. Rev. B* **78**, 195424 (2008).

[11] Xiao, D., Chang, M.-C. & Niu, Q. Berry phase effects on electronic properties. *Rev. Mod. Phys.* **82**, 1959–2007 (2010).

[12] Deretzis, I., Calogero, G., Angilella, G. G. N. & La Magna, A. Role of basis sets on the unfolding of supercell band structures: From tight-binding to density functional theory. *Europhys. Lett.* **107**, 27006 (2014).
